# *Leishmania infantum*-specific production of IFN-γ and IL-10 in stimulated blood from dogs with clinical leishmaniosis

**DOI:** 10.1186/s13071-016-1598-y

**Published:** 2016-06-03

**Authors:** Laia Solano-Gallego, Sara Montserrrat-Sangrà, Laura Ordeix, Pamela Martínez-Orellana

**Affiliations:** Departament de Medicina i Cirurgia Animals, Facultat de Veterinària, Universitat Autònoma de Barcelona, Bellaterra, Spain; Hospital Clínic Veterinari, Universitat Autònoma de Barcelona, Bellaterra, Spain

**Keywords:** *Leishmania infantum*, Dog, IFN-γ, IL-10, Disease, Clinical staging

## Abstract

**Background:**

There is limited information available on cytokine profiles in dogs with different degrees of disease severity due to natural infection of *Leishmania infantum*. The aim of this study was to investigate *L. infantum*-specific IFN-γ and IL-10 production in blood from dogs with leishmaniosis at diagnosis and correlate these findings with disease severity, humoral immune response and blood parasitemia.

**Methods:**

Sixty dogs were diagnosed based on physical examination, routine laboratory tests, *L. infantum*-specific antibody levels measured by quantitative ELISA and blood parasitemia by real-time PCR. Heparin whole blood was stimulated with *L. infantum* soluble antigen (LSA) and concanavalin A (ConA) and incubated for 5 days. IFN-γ and IL-10 concentrations were measured in supernatants with sandwich ELISAs.

**Results:**

The majority of dogs (*n* = 36) were classified as LeishVet stage II (moderate disease). The rest of the dogs were classified as stage I (*n* = 10), III (*n* = 10) and IV (*n* = 4). Dogs classified with stage I and IIa presented significantly higher (*P* = 0.02) LSA IFN-γ concentrations, lower (*P* <0.0001) antibody levels and a tendency for lower blood parasitemia (*P* = 0.1) than dogs classified with stages IIb, III or IV while no differences in ConA IFN-γ or IL-10 concentrations were observed among groups. Thirty-five dogs produced significantly higher LSA IFN-γ (mean ± SD: 2320 ± 3960 pg/ml) and ConA IFN-γ (mean ± SD: 7887 ± 7273 pg/ml) when compared with 25 dogs that did not produce detectable LSA IFN-γ but produced ConA IFN-γ (mean ± SD: 4917 ± 5233 pg/ml). IFN-γ producer dogs presented lower (mean ± SD: 5750 ± 14,082 ELISA units (EU), *P* = 0.001) antibody levels and blood parasitemia (mean ± SD:   5 ± 10 parasites/ml, *P* = 0.001) when compared with IFN-γ non-producers (mean ± SD: 19,638 ± 28,596 EU and 1100 ± 5112 parasites/ml), respectively. LSA IL-10 was not detectable in 34 dogs while 49 dogs secreted ConA IL-10 (mean ± SD of 90 ± 103 pg/ml). LSA IFN-γ concentration was negatively correlated with blood parasitemia and antibody levels and positively correlated with ConA IFN-γ and LSA IL-10 concentrations.

**Conclusions:**

The results of this study demonstrate that sick dogs lacking *L. infantum* specific IFN-γ production in stimulated whole blood produce a strong humoral response, have a high blood parasitemia and severe clinical disease. IL-10 does not appear to be a marker of disease severity.

## Background

Species of *Leishmania* are obligate intracellular protozoan parasites that are transmitted by female phlebotomine sandflies. Canine leishmaniosis is a zoonotic disease caused by several species of *Leishmania*. Although other *Leishmania* species have been reported to infect dogs such as *L. major* [1], *L. tropica* and *L. braziliensis* [2–4], *Leishmania infantum* is the most common species in the Mediterranean basin [5, 6]. Dogs are considered the principal reservoir for leishmaniosis in people due to *L. infantum* in the Mediterranean basin, Middle East and South America [5].

Both innate and adaptive immune responses play a role in the outcome of *Leishmania* infection. Classically, the major role against the parasite is played by the adaptive immune response, characterized by the balance between T-helper 1 (Th1) and T-helper 2 (Th2) response. Th1 secrete interleukin-2 (IL-2), TNF-alpha and interferon-gamma (IFN-γ) and this response is associated with controlling infection while Th2 cells secrete interleukin-4 (IL-4), interleukin-5 (IL-5), interleukin-10 (IL-10), and transformation growth factor beta (TGF-β) and correlate with disease progression [7, 8].

Dogs show a broad range of clinical manifestations and immune responses [9] in canine leishmaniosis. Canine *L. infantum* infection can manifest as a chronic subclinical infection, self-limiting disease, or non-self-limiting illness [5, 6] as in humans [10]. In addition, several degrees of disease severity are found in dogs ranging from mild disease to severe fatal disease with different clinical outcomes, prognosis and treatment options. Therefore, a clinical staging system is currently used in the clinical setting [6]. The two extremes of this clinical spectrum are characterized by: (1) “Resistant” healthy dogs with a protective CD4^+^ T-cell-mediated immune response characterized by production of Th1 cytokines-like such as IFN-γ, IL-2 and TNF-α, which induce anti-*Leishmania* activity by apoptosis of parasites in macrophages via nitric oxide (NO) metabolism [11], and (2) sick dogs which are characterized by a marked humoral immune response, absent or reduced cell mediated immunity with high levels of IL- 10 and TGF-β and high parasite burden, which is detrimental to the animal [5, 12].

Unfortunately, there are few and poorly standardized assays to evaluate T-cell mediated immunity responses [13] such as leishmanin skin test [14, 15] and lymphocyte proliferation [16, 17] in the dog. The results of cytokine profiles in canines with *L. infantum* infection are frequently limited and fragmentary [9]. Cytokines including IFN-γ and IL-10 have been studied in several organs with different techniques in dogs with poorly defined clinical classification and different states of infection [5, 9]. In addition, cytokines have been mainly investigated in vaccinated [18] or in experimentally-infected dogs [17] but limited information is available in sick dogs with different degrees of disease severity due to *L. infantum* natural infection [17, 19]. The aim of this study was to investigate *L. infantum*-specific IFN-γ and IL-10 production in stimulated blood in dogs with clinical leishmaniosis at the time of diagnosis and correlate these with disease severity, the humoral immune response and blood parasitemia.

## Methods

### Dogs

Sixty dogs with clinical leishmaniosis were prospectively enrolled from January 2014 to February 2016 at the time of diagnosis. The dogs were from different Catalonian and Balearic veterinary centers from Spain: *Fundació Hospital Clínic Veterinari* (Bellaterra, Barcelona), *Hospital Ars Veterinaria* (Barcelona), *Hospital Mediterrani Veterinaris* (Reus, Tarragona), *Consultori Montsant* (Falset, Tarragona) and *Hospital Mon Veterinari* (Manacor, Mallorca)*.* The diagnosis of canine leishmaniosis was made based on the results of the physical examination, a complete blood count (CBC) using Siemens Advia 120 Haematology System (Siemens Healthcare GmbH, Germany), a biochemical profile including creatinine, urea, total proteins, ALT and total cholesterol by Olympus AU400 Chemistry Analyzer (CLIAwaived, USA), protein serum electrophoresis by Hydrasys® (Sebia Electrophoresis, USA), urinalysis with urinary protein/creatinine ratio (UPC) and quantitative serology for the detection of *L. infantum* specific antibodies by means of a serial dilution in house ELISA [20–22]. Cytological evaluation of any lesion or cutaneous histology and/or immunohistochemistry for *Leishmania* was also performed in some cases as described elsewhere when needed [23]. In addition, DNA was extracted from blood samples and *L. infantum* real-time PCR (RT-PCR) was performed with an absolute quantification as previously described [22, 24]. Dogs were classified in four clinical stages (stage I-mild disease, II-moderate disease including substages IIa and IIb, III-severe disease and IV-very severe disease) at the time of diagnosis as previously described [6].

### Whole blood assay

The leucocyte concentration including lymphocyte and neutrophil concentrations was very similar between dogs measured by a CBC. Heparinized blood was diluted to a ratio of 1:10 with Rosewell Park Memorial Institute (RPMI) 1640 medium with stable glutamine and 25 mM hepes (Biowest®, USA) supplemented with 60 μg/ml of penicillin, 100 μg/ml streptomycin (Life Technologies™, USA) and 10 % Fetal Bovine Serum Premium South America Origin (Biowest®, USA). Five hundred μl of heparinized blood was mixed with 4.5 ml of complete medium as described above per each well and incubated in 12-well flat bottom plastic culture plates 3596 (Costar® Corning, NY, USA). Three different treatment conditions were established: (i) medium alone; (ii) medium with *L. infantum* soluble antigen (LSA) at a concentration of 10 μg/ml provided by Dr. Cristina Riera (*Facultat de Farmacia*, *Universitat de Barcelona*); and (iii) medium with mitogen concanavalin A (ConA) (100 mg Medicago®, Sweden) at a concentration of 10 μg/ml. The plates were incubated for 5 days at 37 °C in 5 % of CO_2_ air. Following incubation, blood was centrifuged at 300 g for 10 minutes and the supernatant was collected and stored at -80 °C until used.

### Sandwich ELISAs for the determination of IFN-γ and IL-10

Cytokine analysis of IFN-γ (*n* = 60) and IL-10 (*n* = 53) were performed according to the manufacturer’s instructions (DuoSet® ELISA by Development System R&D^TM^, UK) using 96 well cell flat bottom plates (Costar® Corning, USA). Slight modifications were done for the IFN-γ and IL-10 ELISAs. The standard curve for IFN-γ started with 8000 pg/ml and two-fold dilutions were made until 62.5 pg/ml concentration. Standard curve for IL-10 started with 2000 pg/ml and two-fold dilutions were made until 15.6 pg/ml concentration was obtained. Supernatants treated with ConA were diluted 1:1 with reagent diluent. Duplicates of all supernatants studied were performed in all ELISAs. Optical density was measured with an ELISA reader (Anthos 2020) at wavelength of 450 nm. The standard curve for each cytokine was calculated using a computer generated four parameter logistic curve-fit with program MyAssays (http://www.myassays.com/). Plates were repeated when R^2^-value of standard curve was below 0.98. Dogs were classified as IFN-γ producers when *L. infantum* specific IFN-γ concentration was detectable after subtracting medium alone. Dogs were classified as IFN-γ non-producers when *L. infantum* specific IFN-γ concentration subtracting medium alone was at not detectable levels. IL-10 detectable concentrations were considered after subtracting medium alone for LSA and ConA stimulations.

### Statistical analysis

The statistical analysis was performed using the SPSS 22.0 for Windows software (SPSS Inc., USA). A non-parametric Mann-Whitney U-test was used to compare groups. A non-parametric Wilcoxon signed-rank test was used to compare paired continuous variables. The Spearman’s correlation was used to evaluate differences in cytokine production, the level of antibodies and blood parasitemia of the dogs studied. Differences were considered significant with a 5 % significance level (*P* < 0.05).

## Results

### Description of clinical data of dogs, clinical staging and specific *L. infantum* IFN-γ response classifications

Both sexes were represented by 25 females and 35 males. The median of age was four years with a range from five months to 12 years. Forty-four purebred dogs belonging to more than 25 breeds and 16 mixed breed dogs were included.

Based on clinicopathological findings and serological tests, the majority of dogs (*n* = 36) were classified as LeishVet stage II (moderate disease) and this stage was further divided into stage IIa (*n* = 27) and stage IIb (*n* = 9). The rest of the dogs were classified as stage I (*n* = 10), stage III (*n* = 10) and stage IV (*n* = 4). All dogs in stage I were diagnosed with papular dermatitis due to *L. infantum* and presented negative to low positive antibody levels. The rest of dogs presented typical clinicopathological findings and mainly medium to high positive antibody levels. The results of signalment, serology, clinical signs and laboratory abnormalities observed in dogs with clinical leishmaniosis classified in four clinical stages are shown in Table 1.

**Table 1:** Clinicopathological findings in dogs with clinical leishmaniosis based on LeishVet clinical staging [6]

Clinical staging	Gender/median [range] of age in months	Serology	Clinical signs (number of dogs/total number of dogs, %)	Laboratory findings (number of dogs/total number of dogs, %)
I-mild disease (*n* = 10)	5 females and 4 males/15.2 [5–40]	Negative to low positive antibody levels	Papular dermatitis (10/10, 100 %)	No abnormalities observed
IIa-moderate disease (*n* = 27)	11 females and 16 males/51 [5–153]	Low to high positive antibody levels	Cutaneous lesions (20/27, 74 %): • Exfoliative dermatitis (9/27, 33 %) • Ulcerative dermatitis (9/27, 33 %) • Alopecia (7/27, 26 %) • Nodular dermatitis (3/27, 11 %) • Papular dermatitis (2/27, 7 %) • Ischemic dermatopathy (1/27, 4 %) Lymphadenomegaly (19/27, 70 %) Weight loss (8/27, 30 %) Fever (5/27, 19 %) Ocular lesions (4/27, 15 %): • Conjunctivitis (3/27, 11 %) • Blepharitis (1/27, 4 %) Lameness (4/27, 15 %) Vomiting and/or diarrhea (2/27, 7 %) Inspiratory dyspnea (1/27, 4 %) Masseter myositis (1/27, 4 %) Muscle atrophy (1/27, 4 %) Epistaxis (1/27, 4 %)	Mild to moderate normocytic normochromic non-regenerative anemia (8/26, 31 %; 3/26, 12 %, respectively) Mild leukocytosis with mature neutrophilia (2/26, 8 %) Mild to moderate thrombocytopenia (2/26, 8 %) Mild leucopenia with lymphopenia and neutropenia (2/26, 8 %) Hyperproteinemia (13/27, 48 %) Hypergammaglobulinemia (14/27, 52 %) Hyperbetaglobulinemia (15/27, 55 %) Hypoalbuminemia (6/26, 23 %)
IIb-moderate disease (*n* = 9)	2 females and 9 males/43 [9–147]	Medium to high positive antibody levels	Cutaneous lesions (7/9, 77 %): • Exfoliative dermatitis (3/9, 33 %) • Ulcerative dermatitis (3/9, 33 %) • Alopecia (2/9, 22 %) Lymphadenomegaly (6/9, 67 %) Weight loss (5/9, 56 %) Ocular lesions (4/9, 44 %): • Conjunctivitis (2/9, 22 %) • Bilateral blepharitis (2/9, 22 %) Vomiting and/or diarrhea (3/9, 33 %) Lethargy (2/9, 22 %) Lameness and articular pain (1/9, 33 %) Polyuria and polydipsia (1/9, 11 %)	Mild to moderate normocytic normochromic non-regenerative anemia (6/9, 67 %; 2/9, 22 %, respectively) Mild leukocytosis with mild mature neutrophilia (2/9, 22 %) Lymphopenia (3/9, 33 %) Hyperproteinemia (7/9, 77 %) Hypergammaglobulinemia (9/9, 100 %) Hyperbetaglobulinemia (6/9, 66 %) Hypoalbuminemia (6/9, 66 %)
III-severe disease (*n* = 10)	5 females and 4 males/85 [37–132]	Medium to high positive antibody levels	Lymphadenomegaly (7/10, 70 %) Cutaneous lesions (6/10, 60 %): • Exfoliative dermatitis (4/10, 40 %) • Alopecia (1/10, 10 %) • Ulcerative dermatitis (2/10, 20 %) Weight loss (5/10, 50 %) Ocular lesions (3/10, 30 %): • Bilateral blepharitis (1/10, 10 %) • Conjunctivitis (1/10, 10 %) • Superficial keratitis (1/10, 10 %) Lethargy and anorexia (2/10, 20 %) Vomiting and/or diarrhea (2/10, 20 %) Splenomegaly (2/10, 20 %) Lameness and articular pain (1/10, 10 %) Muscle atrophy (1/10, 10 %) Epistaxis (1/10, 11 %) Polyuria and polydipsia (1/10, 10 %) Fever (1/10, 10 %) Chronic weakness (1/10, 10 %)	Mild normocytic normochromic non-regenerative anemia (6/9, 67 %) Moderate regenerative anemia (2/9, 22 %) Thrombocytosis (1/9, 11 %) Mild thrombocytopenia (1/9, 11 %) Mild leukocytosis with mature neutrophilia (2/9, 22 %) Mild leukopenia with lymphopenia and neutropenia (1/9, 11 %) Lymphopenia (1/9, 11 %) Mild lymphocytosis (1/9, 11 %) Hyperproteinemia (8/10, 80 %) Hypergammaglobulinemia (9/10, 90 %) Hyperbetaglobulinemia (9/10, 90 %) Hypoalbuminemia (9/10, 90 %) Proteinuria (UPC) (10/10, 100 %)
IV-very severe disease (*n* = 4)	2 females and 2 males/7.5 [23–120]	Medium to high positive antibody levels	Weight loss (3/4, 75 %) Cutaneous lesions (3/4, 75 %): • Exfoliative dermatitis (2/4, 50 %) • Ulcerative dermatitis (1/4, 25 %) • Onicogriphosis (1/4, 25 %) • Ulcerative and nodular blepharitis (1/4, 25 %) Vomiting and/or diarrhea (2/4, 50 %) Lymphadenomegaly (1/4, 25 %) Cachexy (1/4, 25 %) Hypertension (1/4, 25 %) Cardiac insufficiency (1/4, 25 %) Keratoconjunctivitis (1/4, 25 %) Chronic weakness (1/4, 25 %) Paraparesis (1/4, 25 %)	Mild normocytic normochromic non regenerative anemia (1/4, 25 %; 3/4, 75 %, respectively) Thrombocytosis (2/4, 50 %) Lymphopenia (1/4, 25 %) Mild leukocytosis with mature neutrophilia (1/4, 25 %) Total protein (4/4, 100 %) Hypoalbuminemia ((4/4, 100 %) Hypergammaglobulinemia (3/3, 100 %) Hyperbetaglobulinemia (3/3, 100 %) Proteinuria (UPC) (4/4, 100 %) Renal azotemia (3/3, 100 %)

*UPC:* urinary protein creatinine ratio

**Fig. 1:**
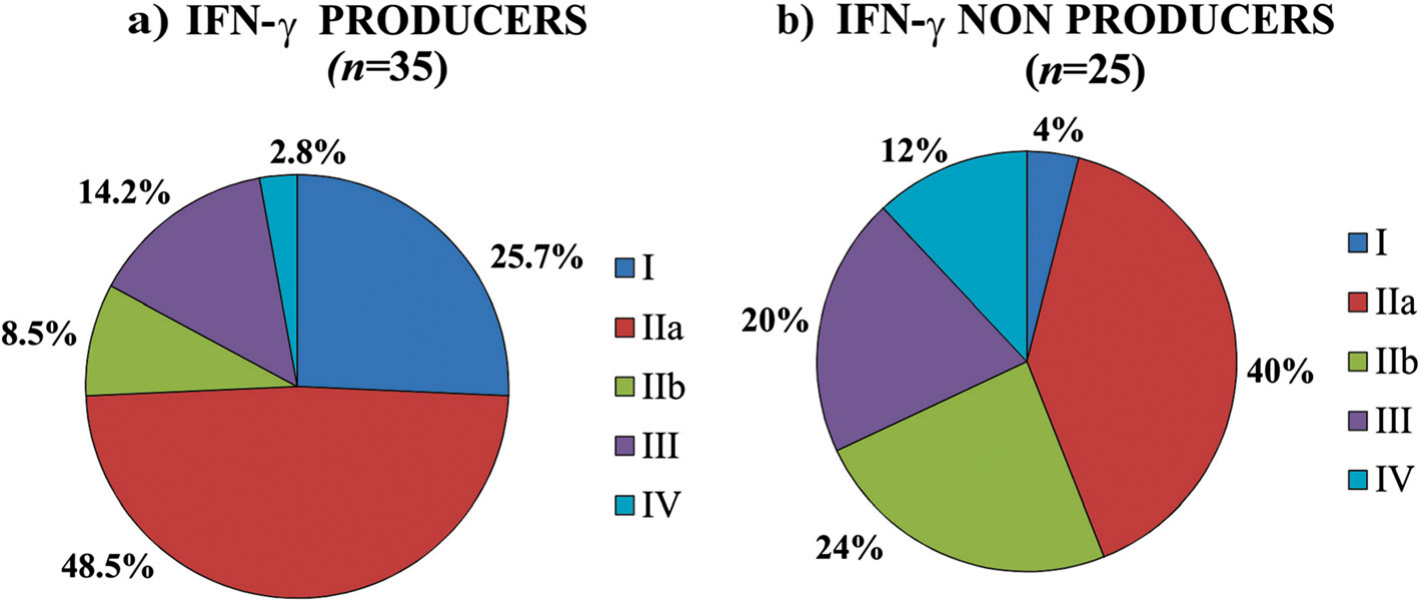
Percentages of sick dogs according with IFN-γ production after LSA stimulation and clinical staging. **a** IFN-γ producers (*n* = 35) and **b** IFN-γ non-producers (*n* = 25)

Thirty-five dogs (58 %) were classified as IFN-γ producers. The clinical staging of these dogs was: nine dogs had stage I (25.7 %), 17 dogs stage IIa (48.5 %), three dogs stage IIb (8.5 %), five dogs stage III (14.2 %) and one dog stage IV (2.8 %) (Fig. 1a). In contrast, 25 dogs did not secrete IFN-γ after LSA stimulation. All 25 dogs (42 %) were classified as IFN-γ non-producers. The clinical staging of IFN-γ non-producers was as follows: one dog (4 %) was classified as stage I; ten dogs as stage IIa (40 %); six dogs as stage IIb (24 %); five dogs as stage III (20 %) and three dogs (12 %) as stage IV (Fig. 1b).

### Antibody response

The results of antibody levels based on clinical staging and IFN-γ classification are shown in Tables 2 and 3. Dogs classified with stage I and IIa presented significantly lower antibodies (Mann-Whitney U-test: Z = -4.37, *P* < 0.0001) (mean±SD: 4050 ± 9947 EU) than dogs classified with stages IIb, III or IV (mean±SD: 23,580 ± 30,308 EU).

**Table 2:** Summary of parameters studied based on clinical staging distribution of sick dogs

Clinical staging	Mean ± standard deviation						
	Number of dogs	IFN-γ LSA (pg/ml)	IFN-γ ConA (pg/ml)	IL-10 LSA (pg/ml)	IL-10 ConA (pg/ml)	PCR (parasite/ml)	ELISA (EU)
I	10	2022 ± 2687^h^	4029 ± 3942^i^	17 ± 18	82 ± 46	3.7 ± 10.4^d^	46 ± 51^a^
IIa	27	1909 ± 4373	7943 ± 7115^j^	8 ± 14	57 ± 50	6 ± 10^e^	5533 ± 11338^b^
IIb	9	539 ± 1231	6777 ± 6068	7 ± 14	144 ± 130	101 ± 285^f^	23,287 ± 27636
III	10	423 ± 629	8004 ± 7914^k^	15 ± 19	94 ± 167	20 ± 26^g^	12,416 ± 20,227^c^
IV	4	94 ± 187	792 ± 350	5 ± 9	77 ± 112	6337 ± 12501	52,151 ± 44,365
Total	60	1354 ± 3220	6649 ± 6618	10 ± 15	83 ± 102	458 ± 3293	11,537 ± 22,240

Parameters under study were evaluated in relationship to clinical staging and letters from ^a^ to ^b^ correspond to statistically significant findings

Results for ELISA units ^a^I < IIa, IIb, III, IV (Mann-Whitney U-test: *Z* = -3.7, *P* < 0.0001); ^b^IIa > I (Mann-Whitney U-test: *Z* = -4.4, *P* < 0.0001) and IIa < IIb, IV (Mann-Whitney U-test: *Z* = -2.7, *P* = 0.002); ^c^III < IV (Mann-Whitney U-test: *Z* = -2.1, *P* = 0.036)

Results for PCR ^d^I < IV (Mann-Whitney U-test: *Z* = -2.8, *P* < 0,002); ^e^IIa < IV (Mann-Whitney U-test: *Z* = -3.1, *P* < 0.0001); ^f^IIb < IV (Mann-Whitney U-test: *Z* = -2.2, *P* = 0.028); ^g^ III < IV (Mann-Whitney U-test: *Z* = -2.2, *P* = 0.024)

Results for IFN-γ LSA ^h^I > IIb, III, IV (Mann-Whitney U-test: *Z* = -2.1, *P* < 0.035)

Results for IFN-γ ConA ^i^I>IV (Mann-Whitney U-test: *Z* = -2.1, *P* = 0.036); ^j^IIa > IV (Mann-Whitney U-test: *Z* = -2.7, *P* = 0.003); ^k^III > IV (Mann-Whitney U-test: *Z* = -2.5, *P* = 0.008)

*Abbreviations: LSA L. infantum* soluble antigen, *ConA* concavalin A

**Table 3:** Summary of parameters based on IFN-γ producer *versus* IFN-γ non-producer dogs

Clinical staging	IFN-γ producers (mean ± standard deviation)					IFN-γ non-producers (mean ± standard deviation)				
	Number of dogs	IFN-γ LSA (pg/ml)	IL-10 LSA (pg/ml)	PCR (parasite/ml)	ELISA (EU)	Number of dogs	IFN-γ LSA (pg/ml)	IL-10 LSA (pg/ml)	PCR (parasite/ml)	ELISA (EU)
I	9	2247 ± 2749	18 ± 20	4.1 ± 11	50 ± 53	1	0 ± 0	15 ± 0	0 ± 0	13 ± 0
IIa	17	3031 ± 5240	12 ± 16	2 ± 4	2660 ± 7140	10	0 ± 0	3 ± 8	12 ± 14	10,418 ± 15,420
IIb	3	1616 ± 1860	14 ± 23	0.1 ± 0.1	14,373 ± 15,473	6	0 ± 0	3.3 ± 8	162 ± 360	27,744 ± 32,476
III	5	846 ± 665	29 ± 18	12 ± 19	7755 ± 5590	5	0 ± 0	0 ± 0	29 ± 31	17,077 ± 28,897
IV	1	375 ± 0	19 ± 0	35 ± 0	73,701 ± 0	3	0 ± 0	0 ± 0	8438 ± 14,419	44,967 ± 51,408
Total	35	2320 ± 3961^a^	16 ± 18^b^	5 ± 10	5750 ± 14,082	25	0 ± 0	2.4 ± 7	1100 ± 5112^c^	19,638 ± 28,596^d^

Statistical study was performed to compare IFN-γ producers *versus* non-IFN-γ producers. Letters a to d correspond to statistically significant differences

Results for IFN-γ LSA, IFN-γ^a^ producers > non-IFN-γ producers (Mann-Whitney U-test: *Z* = -6.7, *P* < 0.0001)

Results for IL-10 LSA, IFN-γ^b^ producers > non-IFN-γ producers (Mann-Whitney U-test: *Z* = -3.60, *P* < 0.0001)

Results for PCR, IFN-γ^c^ producers< non-IFN-γ producers (Mann-Whitney U-test: *Z* = -3.2, *P* = 0.001)

Results for ELISA units, IFN-γ^d^ producers< non-IFN-γ producers (Mann-Whitney U-test: *Z* = -3.2, *P* = 0.001)

*Abbreviations: LSA L. infantum* soluble antigen

IFN-γ producer dogs (mean ± SD: 5750 ± 14,082 EU) showed statistically significant lower levels of antibodies (Mann-Whitney U-test: *Z* = -3.23, *P* = 0.001) when compared with IFN-γ non-producer dogs (mean ± SD: 19,638 ± 28,596 EU) (Table 2).

### Blood parasite load by real-time PCR

The results of blood parasitemia based on clinical staging and IFN-γ classification are shown in Tables 2 and 3. Blood PCR was positive in 40 dogs (66.6 %) out of 59 dogs. In addition, dogs classified with stage I and IIa group presented a tendency (Mann-Whitney U-test: *Z* = -1.53, *P* = 0.1) for lower blood parasitemia (5.3 ± 10.2 parasites/ml) than dogs classified with stages IIb, III or IV group (1255 ± 5464 parasites/ml).

Interestingly, 14 IFN-γ producer dogs and five IFN-γ non-producers were negative by PCR at the time of diagnosis. Twenty-one (60 %) out of 35 IFN-γ producer dogs were positive by PCR at the time of diagnosis. Nineteen (76 %) out of 25 IFN-γ non-producer dogs were positive to PCR at the time of diagnosis. IFN-γ producer dogs presented a statistically significant lower parasitemia (Mann-Whitney U-test: *Z* =-3.22, *P* = 0.001) when compared with IFN-γ non-producers (Table 3).

### IFN-γ

The results of ConA and *L. infantum* specific IFN-γ concentrations based on clinical staging and IFN-γ classification are shown in Tables 2 and 3. IFN-γ concentrations in supernatants of blood stimulated with LSA and ConA from all dogs studied were (mean ± SD) 1354 ± 3220 pg/ml and 6649 ± 6618 pg/ml, respectively. A significantly higher concentration of IFN-γ on ConA stimulated blood was found when compared with LSA stimulated blood (Wilcoxon signed-rank test: *Z* = -6.15, *P* <0.0001). Dogs classified with stage I and IIa group presented significantly higher (Mann-Whitney U-test: *Z* = -2.32, *P* = 0.02) LSA IFN-γ concentrations (mean±SD: 1939 ± 3952 pg/ml) than dogs classified with stages IIb, III or IV group (mean±SD: 411 ± 862 pg/ml) while no differences in ConA IFN-γ concentrations were observed among these two groups.

IFN-γ producer dogs (*n* = 35) showed a significantly higher (Mann-Whitney U-test: *Z* = -6.78, *P* < 0.0001) LSA IFN-γ concentration (mean ± SD of 2320 ± 3961 pg/ml) when compared with IFN-γ non-producers (*n *= 25) (mean ± SD of 0 ± 0 pg/ml). The mean ± SD of ConA IFN-γ concentration from IFN-γ producer dogs was 7887 ± 7273 pg/ml while the mean ± SD of ConA IFN-γ concentration from IFN-γ non-producers was 4917 ± 5233 pg/ml. Statistical differences were found between IFN-γ producers and non-producers for IFN-γ concentration after stimulation with ConA (Mann-Whitney U-test: *Z* = -2.04, *P* = 0.041).

### IL-10

The results of ConA and *L. infantum* specific IL-10 concentrations based on clinical staging and IFN-γ classification are shown in Tables 2 and 3. The mean ± SD of IL-10 concentrations in supernatants of blood stimulated with LSA and ConA from all dogs studied was 10 ± 15 pg/ml and 83 ± 102 pg/ml, respectively. A significantly higher concentration of IL-10 in ConA stimulated blood was found when compared with LSA stimulated blood (Wilcoxon signed-rank test: *Z* = -5.62, *P* < 0.0001). Moreover, 34 out of 53 dogs (64 %) did not produce IL-10 after LSA stimulation (0 pg/ml) while the rest of the dogs (*n* = 19; 36 %) produced significantly higher concentrations (Mann-Whitney U-test: *Z* = -6.46, *P* < 0.0001) of IL-10 (mean ± SD of 27.1 ± 13.3 pg/ml). Only four dogs did not produce IL-10 after ConA stimulation. Statistical analysis showed significantly higher concentrations of IL-10 in IFN-γ producer dogs after LSA stimulation when compared with IFN-γ non-producers (Mann-Whitney U-test: *Z* = -3.60, *P < *0.0001). No significant differences in IL-10 concentrations were observed after ConA stimulation between IFN-γ producers and IFN-γ non-producers.

Concentration of IL-10 from dogs classified with stages I and IIa group (10 ± 15 pg/ml) did not present significant differences when compared with dogs classified with stages IIb, III and IV group (10 ± 16 pg/ml). No differences in ConA IL-10 concentrations were observed between stage I and IIa group and stages IIb, III and IV group.

### Correlation between parameters studied

The correlations among parameters studied are listed in Table 4.

**Table 4:** Correlation between IFN-γ concentration after LSA stimulation and the level of antibodies, blood parasitemia and cytokine production

Parameter (units)	Spearman’s correlation coefficient (r_S_)	*P*-value
*L. infantum* specific antibody levels (EU)	-0.391	0.002
Blood parasitemia (parasites/ml)	-0.413	0.001
IFN-γ concentration after ConA stimulation (pg/ml)	0.311	0.016
IL-10 concentration after LSA stimulation (pg/ml)	0.585	< 0.0001
IL-10 concentration after ConA stimulation (pg/ml)	-0.81	0.562

*Abbreviations: LSA L. infantum* soluble antigen, *ConA* concavalin A

IFN-γ concentration after LSA stimulation was significantly negatively correlated with blood PCR and antibody levels as well as positively correlated with IFN-γ concentration after ConA stimulation and IL-10 concentration after LSA stimulation. No further significant correlations were found with IL-10 production.

## Discussion

In the present study, we demonstrated, for the first time, that dogs with several clinical stages of leishmaniosis presented differences in *L. infantum* specific cytokine profiles in stimulated blood. Interestingly, more than half of the dogs with clinical leishmaniosis showed a marked *L. infantum* specific IFN-γ production. Canine clinical leishmaniosis is classically characterized by a reduced or absent *L. infantum* specific T-cell mediated immunity [25]. In contrast, *Leishmania*-specific IFN-γ production in stimulated blood in dogs [26, 27] and other mammals such as rodents and humans have been associated with a resistant phenotype or with animals able to control infection [9]. However, more recent studies have already highlighted that T-cell mediated immunity might also be observed in dogs with clinical illness [28, 29] in agreement with the present results.

It is also important to remark that the majority of dogs (58 %) that were *L. infantum* specific IFN-γ producers were also classified with the lower clinical stages and were mainly classified as having stage I or stage IIa [6]. Furthermore, significant higher *L. infantum* IFN-γ concentration was noted between stage I and IIa group and stage IIb, III and IV group. In addition, IFN-γ producer dogs presented lower antibody levels as well as lower blood parasitemia when compared with IFN-γ non-producers. Moreover, significant negative correlations were found between LSA IFN-γ concentration and antibody and blood parasitemia. Similar findings were observed in Brazil where pro-inflammatory cytokines such as IFN-γ and TNF-α were highly expressed in the spleen of naturally infected dogs with low parasitism and these cytokines were negatively correlated with the parasite burden in the spleen [29]. An experimental canine model also demonstrated that splenic parasite burdens correlated negatively with *Leishmania*-specific IFN-γ and IL-2 levels, and negatively with leishmanin skin test reactivity [30].

In the present study, 42 % of the dogs were IFN-γ non-producers and these dogs were most likely to be classified in higher clinical stages (stage IIb, III and IV) and also had high specific positive antibody levels and blood parasitemia as previously described in other studies in dogs with severe clinical leishmaniosis [19, 31]. It has been previously demonstrated that as dogs progress to clinical leishmaniosis, they display impaired CD4+ T cell proliferation and IFN-γ production *ex vivo* in response to *L. infantum* antigen [19, 31]. However, the cellular basis and mechanisms for the development of antigen specific T-cell unresponsiveness in canine leishmaniosis are not fully understood. T cell exhaustion is well documented in chronic infections such as parasitic infections in several mammals. T cell exhaustion is defined as antigen-specific effector T cell dysfunction with sustained expression of inhibitory receptors, including programmed cell death protein 1 (PD-1) and decreased effector cytokine production such as IFN-γ [32, 33]. Interestingly, T cell exhaustion was significantly associated with a four-fold increase in the population of T cells (both CD4^+^ and CD8^+^ T cells) with PD-1 in a study comparing healthy non-infected control and sick foxhounds dogs with clinical leishmaniosis [31]. This was in conjunction with an absent lymphocyte proliferation and decreased peripheral blood mononuclear cells ( PBMC) *L. infantum* specific IFN-γ production. These authors also demonstrated that PD-1 mediated exhaustion influenced macrophage-reactive oxygen intermediate production enabling the control of *Leishmania* infection [31]. We believe that T-cell exhaustion is probably the mechanism that explains *L. infantum* antigen T-cell unresponsiveness in our patients with moderate to very severe disease. Further studies should elucidate if PD-1 is diversely expressed in dogs in different clinical stages as the ones described in the present study.

Furthermore, in this study we have demonstrated that there were significant differences in IFN-γ production after stimulation with ConA among clinical stages as well as between IFN-γ producer and non-producer dogs. In stage IV-very severe disease, the IFN-γ concentration after stimulation with ConA was significantly lower than in other less severe clinical stages (I, IIa and III) and the same tendency was noted with dogs in stage IIb. In addition, IFN-γ non-producer dogs showed significantly lower IFN-γ concentration after stimulation with ConA when compared with IFN-γ producer dogs. These findings demonstrate that dogs with very severe disease are in more T cell anergy than dogs with less severe clinical stages. This was previously demonstrated by means of mitogenic lymphocyte proliferation such as conA in dogs with clinical leishmaniosis [34, 35].

Interestingly, our findings of IL-10 are not in agreement with the majority of studies on human visceral leishmaniosis [36, 37] or other studies reported in dogs with clinical leishmaniosis where a low or absent IFN-γ production was found in tandem with increased production of IL-10 [19, 29]. An experimental canine model also demonstrated that splenic parasite burdens correlated positively with *Leishmania*-specific IL-10 levels [30]. Interestingly, a recent study carried out in Ethiopia in humans with visceral leishmaniosis due to *L. donovani* showed that *Leishmania* stimulated whole blood cells produce low or levels below the detection limit of IFN-γ and IL-10 [38] in agreement with the results of IFN-γ non-producer dogs of this study. In the present study, IFN-γ producer dogs secreted higher levels of IL-10 concentration after LSA stimulation when compared with IFN-γ non-producers. The reasons for these findings are difficult to explain but it could be because after LSA stimulation, IFN-γ non-producer dogs might be less responsive to other cytokines including IL-10. Another possibility could be that IL-10 is produced in IFN-γ producer dogs as a negative feedback to control proinflammatory cytokines as described in human mucocutaneous leishmaniosis [39]. IL-10 can counterbalance the proinflammatory effect of IFN-γ, leading to an adequate regulation of immune responses. In addition, it is important to remark that a high proportion of dogs did not produce IL-10 and that, in general, IL-10 concentration was extremely much lower than IFN-γ concentration. Moreover, IL-10 concentration after LSA stimulation was not associated with disease severity. Therefore, IL-10 does not appear to be a marker of disease severity.

Suppressed cell-mediated immunity in clinical canine leishmaniosis as a result of the inability of PBMCs to respond to *Leishmania* antigen, is thought to underlie the progressive nature of this disease [9]. In the present study, we reported the ability of a whole blood IFN-γ release assay to detect dogs with clinical leishmaniosis with moderate to severe disease as previously reported in humans with visceral leishmaniosis due to *L. donovani* [40–43]. To date, there are few and poorly standardized assays to evaluate *L. infantum* specific T-cell mediated immunity responses in dogs [13] such as the leishmanin skin test [14, 15], lymphocyte proliferation assays [16, 17] and cytokine profiles in stimulated PBMC or blood from dogs [44, 45]. This type of *L. infantum* specific whole blood assay has been described before in *Leishmania* vaccinated dogs [44, 45]. However, to the best knowledge of the authors, this is the first time, that this whole blood assay has been performed in dogs with clinical leishmaniosis at different clinical stages. In human visceral leishmaniosis, whole blood assay that uses soluble* Leishmania* antigen have shown advantages over the leishmanin skin test, in terms of higher specificity and better correlation with surrogate markers of exposures to *L. donovani* [43]. Furthermore, in humans, CD4^+^ T cells were found to be crucial for and the main source of the IFN-γ production in *Leishmania* stimulated whole blood cultures [42] and we postulated that the same occurs in canines. We believe as described in human visceral leishmaniosis [43], that this assay is a promising tool to evaluate cellular immune responses in dogs in endemic areas. Research in this area is essential for the development of potential immunological and epidemiological tools for both canine and human leishmaniosis.

Our findings confirmed that canine leishmaniosis manifests in a wide spectrum of clinical illness as well documented elsewhere in clinical cases [46], studies [47] and guidelines [6, 48]. The majority of dogs (60 %) were classified as LeishVet stage II (moderate disease) followed by stages I (16 %), III (16 %) and IV (6.6 %). In addition, stage IIa (45 %) was the most frequently diagnosed clinical stage in the present study. The present results gave a good picture of the current presentation of clinical canine leishmaniosis at least in the north-east of Spain in client-owned dogs based on clinical stages. Our findings are likely to be similar to those present in other Mediterranean countries such as Portugal, France or Italy. It is important to highlight that the majority of the dogs studied did not have evidence of renal disease and, therefore, they had a good prognosis if managed with anti-*Leishmania* treatment.

## Conclusions

The results of this study demonstrate that sick dogs lacking *L. infantum* specific IFN-γ production in stimulated whole blood produce a strong humoral response, have a high blood parasitemia and severe clinical disease. In contrast, IL-10 does not appear to be a marker of disease severity. Pinpointing the precise immune responses associated with canine leishmaniosis will help to advance treatment and the development of a preventative strategy.

## Abbreviations

ALT, alanine aminotransferase; CBC, complete blood cell count; CD4, cluster of differentiation 4; ConA, concanavalin A; DNA, deoxyribonucleic acid; ELISA, enzyme-linked immunosorbent assay; EU, ELISA units; IFN-γ, interferon-gamma; IL-10, interleukin-10; IL-2, interleukin-2; IL-4, interleukin-4; IL-5, interleukin-5; LSA, *L. infantum* soluble antigen; NO, nitric oxide; PBMC, peripheral blood mononuclear cells; PD-1, programmed cell death protein 1; RPMI-1640, Roswell Park Memorial Institute 1640 medium; rt-PCR, real time PCR; TGFβ, transforming growth factor beta; Th1, type 1 T helper lymphocytes; Th2, type 2 T helper lymphocytes; TNFα, Tumor Necrosis Factor-alpha; UPC, urinary protein/creatinine ratio.
