# Erratum to: *Leishmania infantum*-specific production of IFN-γ and IL-10 in stimulated blood from dogs with clinical leishmaniosis

**DOI:** 10.1186/s13071-016-1751-7

**Published:** 2016-08-22

**Authors:** Laia Solano-Gallego, Sara Montserrat-Sangrà, Laura Ordeix, Pamela Martínez-Orellana

**Affiliations:** 1 Departament de Medicina i Cirurgia Animals, Facultat de Veterinària, Universitat Autònoma de Barcelona, Bellaterra, Spain; 2 Hospital Clínic Veterinari, Universitat Autònoma de Barcelona, Bellaterra, Spain

## Erratum

Unfortunately, the original version of this article [1] contained an error. The name of the author Sara Montserrat-Sangrà was incorrectly spelt as Sara Montserrrat-Sangrà. The correct spelling is included here, and has been updated in the original article. The publisher would like to apologize for any inconvenience this oversight may have caused.
